# CKD in a Young Man with Nephrogenic Diabetes Insipidus

**DOI:** 10.34067/KID.0000000647

**Published:** 2025-03-27

**Authors:** Isaac Teitelbaum

**Affiliations:** University of Colorado Anschutz Medical Campus School of Medicine, Aurora, Colorado

**Keywords:** CKD, diabetes insipidus

## Abstract

**Figure:**
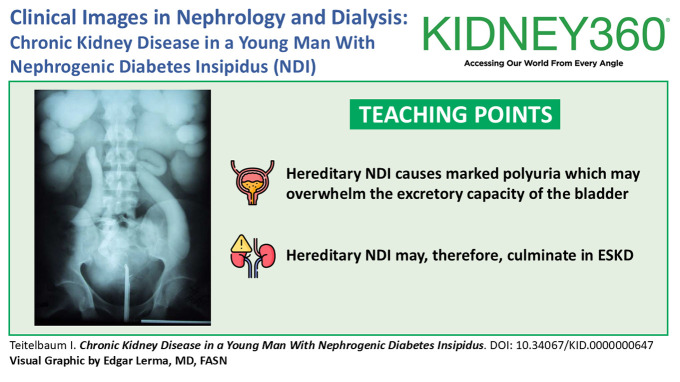

## Case Description

**Figure 1:**
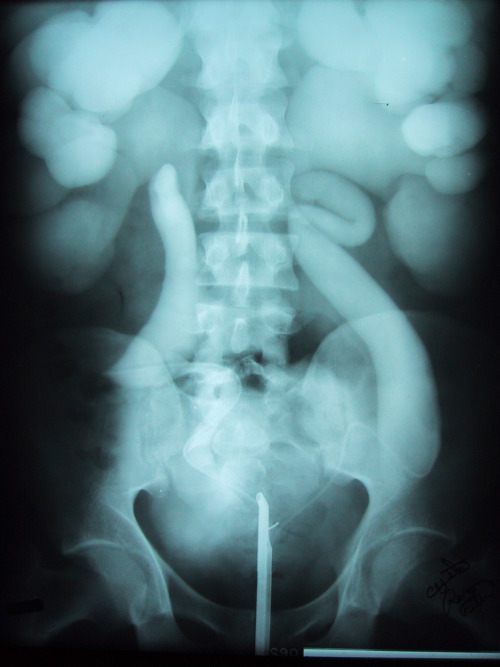
Retrograde pyelogram demonstrating bilateral symmetric severe hydroureteronephrosis with dilation and ballooning of calyces, cortical thinning, and tortuous course of the ureters.

A 24-year-old man presented with CKD, with serum creatinine in the range of 3 mg/dl (the exact value is unavailable, as this case dates to 1985 and all official medical records have since been destroyed). He was known to have hereditary nephrogenic diabetes insipidus (NDI), for which he was prescribed hydrochlorothiazide (HCTZ). He was also affected by schizophrenia and was therefore only intermittently compliant with the HCTZ. His urine output ranged from approximately 0.5 to 1 L/h. Retrograde pyelography was performed (Figure 1), demonstrating bilateral symmetric severe hydroureteronephrosis with dilation and ballooning of calyces, cortical thinning, and tortuous course of the ureters. The patient ultimately progressed to ESKD and was treated with incenter hemodialysis before dying approximately 5 years later.

## Discussion

Hereditary NDI is a rare X-linked disorder in which dysfunction of the vasopressin V2 receptor or a defect in the aquaporin 2 water channel results in inability to concentrate the urine, culminating in marked polyuria.^1^ Treatment is targeted at decreasing urine volume with diuretics, for example, HCTZ or amiloride, and inhibiting prostaglandin synthesis (while simultaneously decreasing GFR) with cyclooxygenase inhibitors, for example, indomethacin.^1^ Despite treatment, prolonged marked polyuria may, even in the absence of bladder outlet obstruction, functionally overwhelm the excretory capacity of the bladder, causing upper urinary tract damage, for example, hydronephrosis and hydroureter,^2,3^ similar to that seen in patients with neurogenic bladder or spinal cord injury.^4,5^ Our patient was not treated with cyclooxygenase inhibition; although kidney biopsy to eliminate other causes of CKD could not be performed because of the marked hydronephrosis, we felt it most likely that his CKD was due to the hydronephrosis alone.

## Teaching Points

- Hereditary NDI causes marked polyuria, which may overwhelm the excretory capacity of the bladder.
- Hereditary NDI may, therefore, culminate in ESKD.
